# Overrepresentation of Glutamate Signaling in Alzheimer's Disease: Network-Based Pathway Enrichment Using Meta-Analysis of Genome-Wide Association Studies

**DOI:** 10.1371/journal.pone.0095413

**Published:** 2014-04-22

**Authors:** Eduardo Pérez-Palma, Bernabé I. Bustos, Camilo F. Villamán, Marcelo A. Alarcón, Miguel E. Avila, Giorgia D. Ugarte, Ariel E. Reyes, Carlos Opazo, Giancarlo V. De Ferrari, the Alzheimer's Disease Neuroimaging Initiative, the NIA-LOAD/NCRAD Family Study Group

**Affiliations:** 1 Center for Biomedical Research and FONDAP Center for Genome Regulation, Faculty of Biological Sciences and Faculty of Medicine, Universidad Andres Bello, Santiago, Chile; 2 Faculty of Biological Sciences, Universidad de Concepción, Concepción, Chile; 3 Oxidation Biology Laboratory, The Florey Institute of Neuroscience and Mental Health, The University of Melbourne, Melbourne, Australia; University of Iowa Hospitals & Clinics, United States of America

## Abstract

Genome-wide association studies (GWAS) have successfully identified several risk loci for Alzheimer's disease (AD). Nonetheless, these loci do not explain the entire susceptibility of the disease, suggesting that other genetic contributions remain to be identified. Here, we performed a meta-analysis combining data of 4,569 individuals (2,540 cases and 2,029 healthy controls) derived from three publicly available GWAS in AD and replicated a broad genomic region (>248,000 bp) associated with the disease near the APOE/TOMM40 locus in chromosome 19. To detect minor effect size contributions that could help to explain the remaining genetic risk, we conducted network-based pathway analyses either by extracting gene-wise p-values (GW), defined as the single strongest association signal within a gene, or calculated a more stringent gene-based association p-value using the extended Simes (GATES) procedure. Comparison of these strategies revealed that ontological sub-networks (SNs) involved in glutamate signaling were significantly overrepresented in AD (p<2.7×10^−11^, p<1.9×10^−11^; GW and GATES, respectively). Notably, glutamate signaling SNs were also found to be significantly overrepresented (p<5.1×10^−8^) in the Alzheimer's disease Neuroimaging Initiative (ADNI) study, which was used as a targeted replication sample. Interestingly, components of the glutamate signaling SNs are coordinately expressed in disease-related tissues, which are tightly related to known pathological hallmarks of AD. Our findings suggest that genetic variation within glutamate signaling contributes to the remaining genetic risk of AD and support the notion that functional biological networks should be targeted in future therapies aimed to prevent or treat this devastating neurological disorder.

## Introduction

Alzheimer's disease (AD [MIM 104300]) is the most common neurodegenerative disorder in the human population [1]. Clinically, AD is characterized by a progressive loss of cognitive abilities and memory impairment. At a biological level, it is thought that the presence of extracellular deposits of the β-amyloid peptide (Aβ) and intracellular neurofibrillary tangles composed of hyperphosphorylated Tau protein leads to synaptic loss and neuronal death [1], [2]. Genetically, AD is complex and heterogeneous.[3], [4] A small percentage of AD cases (1–2% of all cases) have an early-onset familial form of presentation, with symptoms appearing before 65 years of age, and most cases are late-onset or “sporadic” with no apparent familial recurrence of the disease [4]. While familial-AD has been associated with mutations in the genes coding for the amyloid precursor protein (APP) and the presenilins (PSEN1 and PSEN2) proteins, the only genetic factor extensively replicated for sporadic AD is the apolipoprotein E-ε4 (APOE-ε4) allele [4]–[6], which is present in ca. 60% of the cases [1], [7]–[9]. However, the APOE-ε4 allele is not causative, since it has been found in individuals that would not develop the disease, suggesting that other genetic contributions remain to be identified.

During the past decade, the scientific efforts focused in identifying these genetic hallmarks reported more than 2,900 Single Nucleotide Polymorphisms (SNP) within ∼ 4,700 genes associated with AD [10] (see also AlzGene.org). More recently, the use of high density DNA genotyping microarrays in genome-wide associations studies (GWAS), combined with powerful statistical procedures, have expanded the search for novel susceptibility loci for the disease [11]. Nevertheless, these genetic approaches currently exhibit some limitations. First, they present a high rate of false positives and require major sample sizes in order to be replicated. In fact, different simulations have shown that authentic associations have only a 26% chance of falling into the top 1,000 p-values in a GWAS [12]. Second, they only examine the association of a single genetic variant at a time, therefore failing in the detection of minor associations that can still be present and confer risk in a cumulative way. Finally, current top hits associated with AD, including the bridging integrator 1 (BIN1) [13], clusterin (CLU) [14], [15], the ATP-binding cassette sub-family A member 7 (ABCA7) [16], the complement component (3b/4b) receptor 1 (CR1) [15], and the phosphatidylinositol binding clathrin assembly protein (PICALM) [14], do not account for the entire genetic contribution of the disease or surpass the risk conferred solely by the APOE-ε4 allele.

Therefore, considering that the etiology of complex diseases might depend on functional protein-protein interaction networks [17], [18], here we performed a meta-analysis followed by network-based pathway analyses on publicly available GWAS in AD and used significant genetic information to identify glutamate signaling as a key ontological pathway of the disease.

## Subjects and Methods

### GWAS datasets included in the analysis

We selected three publicly available GWAS in AD performed on unrelated case-control and familial samples from European-descent populations (Table 1). The GWAS datasets are: i) the Translational Genomics Research Institute (TGen1) study on AD [19], including 829 AD cases and 535 control individuals; ii) the National Institute on Aging - Late Onset Alzheimer's Disease and the National Cell Repository for Alzheimer's Disease (NIA-LOAD/NCRAD) [20], [21], including 5,220 subjects from which we only considered for analysis a subset of 3,689 individuals (1,837 cases and 1,852 controls) that were self-declared non-Hispanic European Americans, passed principal components analyses and had non-missing phenotypes. Given that this subset is composed by familial data, using the provided family trees, we excluded all related controls and kept only one case per family giving a final number of 978 AD cases and 702 controls individuals that were eligible for our study; and iii) the Pfizer Pharmaceutical Company (Pfizer) study on AD [13], including 733 AD cases and 792 control individuals, available only at summary level. The TGen1 data was downloaded from the TGen website (https://www.tgen.org/research/research-divisions/neurogenomics.aspx) and the NIA-LOAD/NCRAD data was retrieved through the database of Genotypes and Phenotypes (dbGaP; http://www.ncbi.nlm.nih.gov/gap) [22] of the National Institute of Health (NIH), under the accession number phs000168.v1.p1. The Pfizer data was gathered from the supplementary information accompanying the original publication [13]. Detailed information regarding recruitment, diagnosis, affection status and age at the time of enrollment can be found in the original studies [13], [19], [21]. Written informed consent was obtained for all participants and prior Institutional Review Board approval was obtained at each participating institution. Additionally, data used in the preparation of this article were obtained from the Alzheimer's Disease Neuroimaging Initiative (ADNI) database (http://adni.loni.usc.edu) as a targeted replication sample. The ADNI was launched in 2003 by the National Institute on Aging (NIA), the National Institute of Biomedical Imaging and Bioengineering (NIBIB), the Food and Drug Administration (FDA), private pharmaceutical companies and non-profit organizations, as a $60 million, 5-year public-private partnership. The primary goal of ADNI has been to test whether serial magnetic resonance imaging (MRI), positron emission tomography (PET), other biological markers, and clinical and neuropsychological assessment can be combined to measure the progression of mild cognitive impairment (MCI) and early Alzheimer's disease (AD). Determination of sensitive and specific markers of very early AD progression is intended to aid researchers and clinicians to develop new treatments and monitor their effectiveness, as well as lessen the time and cost of clinical trials. The Principal Investigator of this initiative is Michael W. Weiner, MD, VA Medical Center and University of California – San Francisco. ADNI is the result of efforts of many co-investigators from a broad range of academic institutions and private corporations, and subjects have been recruited from over 50 sites across the U.S. and Canada. The initial goal of ADNI was to recruit 800 subjects but ADNI has been followed by ADNI-GO and ADNI-2. To date these three protocols have recruited over 1500 adults, ages 55 to 90, to participate in the research, consisting of cognitively normal older individuals, people with early or late MCI, and people with early AD. The follow up duration of each group is specified in the protocols for ADNI-1, ADNI-2 and ADNI-GO. Subjects originally recruited for ADNI-1 and ADNI-GO had the option to be followed in ADNI-2. After QC procedures (See below; Association analysis) the final ADNI sample consisted in a total of 693 individuals, 449 cases (161 AD and 338 MCI Cases) and 194 unrelated controls. A total of 524,993 SNPs were genotyped under the Illumina 610 Quad platform (Table 1).

**Table 1:** Genome-Wide Association Studies main features.

Datasets	Samples	Ethnicity	Cases	Controls	Platform	SNPs
TGen1	1,364	Caucasian	829	535	Affymetrix 500 K GeneChip	1,231,704^a^
NIA-LOAD/NCRAD	1,680	Caucasian	978	702	Illumina 610-Quad	1,245,964^a^
Pfizer	1,525	Caucasian	733	792	Illumina 550 K, 610-Quad	439,113
Total Meta-analysis	4,569	Caucasian	2540	2029	-	1,216,213^b^
ADNI (replication)	693	Caucasian	499^c^	194	Illumina 610-Quad	524,993

a Imputed. ^b^SNPs shared between the 3 studies. ^c^AD + mild cognitive impairment (MCI) individuals.

### Imputation

In order to maximize information on linkage disequilibrium (LD) structure between the studies, the TGen1 and the NIA-LOAD/NCRAD datasets were imputed by comparison with the CEU reference panel (unrelated individuals) from the HapMap III phased data (release 2) [23]. Imputation was carried out using the Markov Chain Haplotyping method implemented in MACH 1.0 following author recommendations [24].

### Association analysis

Quality control (QC) procedures such as minor allele frequency (MAF), Hardy-Weinberg equilibrium (HWE), missing rate per individuals (MIND) and per SNPs (GENO) were performed on the TGen1 and the NIA-LOAD/NCRAD dataset using PLINK v.1.07 (http://pngu.mgh.harvard.edu/purcell/plink/) [25] with threshold values of 0.05, 1×10^−6^, 0.05 and 0.02, respectively. We applied a logistic regression analysis, using an additive model on the imputed datasets data with MACH2DAT [24]. SNPs with r^2^ values less than 0.29 were removed from further analysis. Similarly, in the Pfizer dataset, the standard error (SE) per SNP was estimated from the p-values reported in the study [13]. Briefly, p-values were transformed into the corresponding Z score with the INVNORMAL function implemented in STATA v.10 (StataCorp, College Station, TX), and then the SE was calculated taking the log of the odds ratios (OR) divided by the corresponding Z score. In the ADNI replication dataset, we performed QC and association analysis based on a quantitative trait locus (QTL) method as described previously [26]. MAF, HWE, MIND and GENO QC values of 0.05, 1×10^−6^, 0.1 and 0.1 were applied, respectively. In order to control for population stratification we conducted Principal Component Analysis (PCA) with EIGENSTRAT [27]. After this step, 63 individuals were excluded from further analysis, leaving a total 693 individuals. The QTL association analysis was carried out using an additive genetic linear regression model with PLINK using different co-variables including age at baseline visit, education, gender and APOE status (ε4 allele present or not). Finally, the results for each dataset was assessed for genomic inflation and visualized in Quantile-Quantile (Q-Q) plots using the statistical R (www.r-project.org) [28].

### Meta-analysis

Meta-analysis was performed using the inverse variance method implemented in PLINK v.1.07 [25]. We checked that all statistics values (p-values, OR and SE) for each dataset prior the meta-analysis were computed for the same allele. Annotation of the results was done with the RefSeq Genes for the human genome assembly Build 36.3/Hg18 available at the UCSC Table Browser [29] using own Perl scripts (available upon request). In order to consider a SNP inside a gene we defined a threshold of +/− 5,000 bp relative to the transcription start and end sites. The annotated output of the meta-analysis used for the pathway approach is available in Table S1. PRISMA guidelines were followed (showed in Checklist S1) [30].

### Single-gene p-value generation

Genetic association values with a cut-off threshold of p<0.05, from either the Meta-analysis or the ADNI replication dataset, were transformed into single-gene associations using two independent approaches: i) Selection of a gene-wise p-value (GW) [17], defined as the single strongest association inside a gene; and ii) Calculation of a gene-based association p-value using the extended Simes procedure (GATES), which extract a gene-level association from the combination of the SNPs p-values within a gene. This approach does not relies on genotype or phenotypic data and has been shown to correct type 1 error rates in both simulated and permuted datasets, regardless of the gene size or LD structure [31]. GATES is an open-source tool named Knowledge-Based Mining System for Genome-wide Genetic Studies (KGG; http://http://bioinfo1.hku.hk:13080/kggweb/).

### Functional Protein Association Network (FPAN)

To evaluate single-gene associations in a network context the complete human functional protein association network (FPAN) was retrieved from the STRING 9.0 database (Search Tool for the Retrieval of Interacting Genes/Proteins; http://string-db.org/) [32]. FPAN contains highly curated known and predicted interactions emerging from different evidence channels such as: genomic context, co-expression and curated literature. Raw text-formatted protein-protein functional interactions were downloaded from STRING. To avoid redundancy and false positives, alternative proteins and their interactions were consolidated into one gene using own Perl scripts (available upon request). We kept only the interactions with a combined score >0.7 (provided by STRING), which stand for high confidence interactions. The final FPAN generated was composed by 14,793 nodes (genes) and 229,357 edges (interactions) and was introduced as an input to Cytoscape [33], which is an open source platform for visualizing complex networks that not only allows the integration of additional attribute data (i.e. gene annotations, expression profiles and interactions source and confidence), but also provides a comprehensive set of tools to perform integrated pathways analysis. Thus, the p-values of GW and GATES procedures were introduced as a floating-point attribute into the FPAN (Table S2).

### Sub-networks search (SNs)

SNs search was carried out with the Cytoscape JActive Modules Plug-in [34] with a gene overlap threshold of 50%. JActive modules is designed to detect if a certain group of connected nodes are significantly enriched with a statistical parameter such as the single gene p-value, which in our study comes from either the meta-analysis or the ADNI replication dataset. Briefly, starting from one node a sub-network grows to its connected genes by computing an aggregated score (S) derived from the conversion of the single-gene p-value (if present) into their corresponding z-score (with the inverse normal cumulative distribution function). This score is compared internally with a background distribution created from the scores of 10,000 random modules of the same size in a Monte Carlo procedure. If the aggregated score cease growing above the expected by chance, the algorithm stops and the growing sub-network is reported as a result. As in the original publication, modules with S>3 (3 standard deviation above the mean of randomized scores) and with a size below 50 were considered significant [17]. To acquire a mean S score and standard deviation (SD) for each resulting SN and to confirm that the SN structure (gene members and interactions) and significance remained consistent and replicable, the search was performed 10 times for each analysis (Meta-GW, Meta-GATES, ADNI-GW and ADNI-GATES). Finally, the same procedure was conducted with their corresponding permuted p-values over the entire genes present in the FPAN (Permuted analysis) and without genome wide significant results (p-values <10^−8^), in this case with real and permuted data, respectively (WGW analysis). Statistical differences between permuted and non-permuted analyses were assessed through two-sided t-test.

### Gene Ontology (GO) and KEGG pathway enrichment Analysis

To examine if the structure of significant sub-networks obtained in the Meta or the ADNI replication dataset were biologically meaningful, gene lists of the first 10 significant modules were tested for pathway enrichment using information from Gene Ontology (GO; http://geneontology.org/) [35] and the Kyoto Encyclopedia of Genes and Genomes database (KEGG; http://www.genome.jp/kegg/) [36]. We initially used the ontology structure and annotations using the package Ontologizer [37], only considering categories with less than 500 members to avoid associations to major categories that are less informative (i.e. signaling) and excluding the ones "Inferred by Electronic Annotation" (IEA), from "Reviewed Computational Analysis" (RCA) and with "No biological Data available" (ND), which are characterized by a high rate of false positives [38]. In this case, we used the parent-child-union algorithm to call for overrepresentation adjusting the p-values with a Westfall-Young Single-Step multiple test correction, to avoid additional false positives [39], and considering a GO term significantly over-represented when the adjusted p-value was below 0.01. Similarly, to determine overrepresentation, KEGG pathway enrichment was assessed in the complete set of pathways and components [40], using an hypergeometric test with the phyper function contained in the R statistical package [41].

### Gene expression heatmaps and cluster analysis

To evaluate the expression pattern of genes of interest from the network-based analysis, human gene expression profiles were downloaded from the Allen Brain Atlas (ABA) website (http://www.brain-map.org) [42]. We used the Gene Search web-tool to enter a list of genes arising from the intersection of sub-networks and analyzed their expression profiles through 27 brain regions. We averaged the expression levels from 6 brain donor individuals (ids. H0351.2001, H0351.2002, H0351.1009, H0351.1012, H0351.1015 and H0351.1016) and used the collapseRows R script [43] to generate a gene-wise expression dataset. Expression heatmaps and hierarchical clusters were analyzed using Cluster v.3.0 (http://www.geo.vu.nl/~huik/cluster.htm) and visualized with the aid of JavaTreeView v.1.1.6r2 (http://jtreeview.sourceforge.net) [44]. Identification of genes co-expressed and correlation analyses were performed with Cluster v.3.0, using Euclidean distance in conjunction with centroid linkage algorithms, and a correlation coefficient cutoff of r>0.7 to denote highly correlated gene clusters.

## Results

### Meta-Analysis

**Figure 1:**
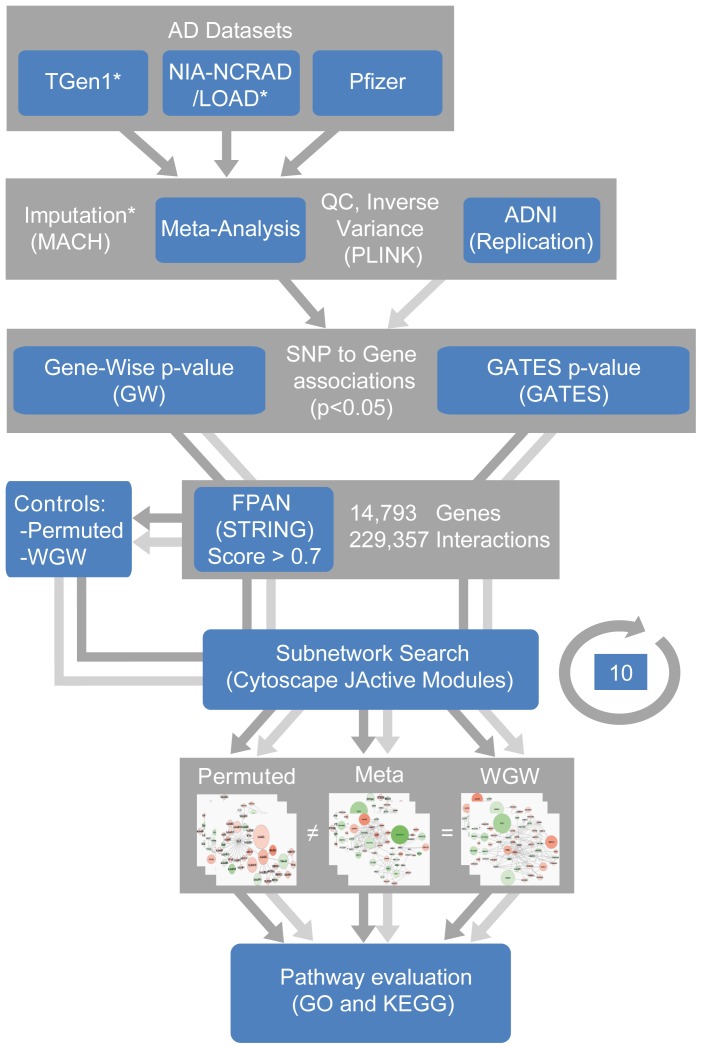
Study strategy. Genotype imputation was carried out in the Tgen1 and NIA-LOAD/NCRAD datasets (asterisk). The meta-analysis was conducted using the inverse variance method in PLINK, after pruning bad genotypes and samples using standard quality control (QC) tests. The ADNI dataset was included for replication following similar QC procedures at this step. Meta-analysis (dark grey arrows) and ADNI associations (light grey arrows) results were annotated and single gene p-values calculated using the Gene-Wise (GW) method or the more stringent GATES procedure (threshold p<0.05). We next introduced this information to FPAN (from STRING database). Module search was performed 10 times, side by side with the permuted data and without genome-wide-significant (WGW) results, which served as internal controls. Significant sub-networks (white squares) were compared and assayed for gene ontology (GO) term and KEGG pathways enrichment to obtain the final overrepresented pathways associated with AD, inside each sub-network. Equal results between Meta and WGW analysis (“ = ”) that could not be obtained with the permuted control (“≠”) were expected.

The complete strategy implemented in the present study is shown in Figure 1. General features of the datasets used for the meta-analysis (TGen1, NIA-LOAD/NCRAD and Pfizer) and for the targeted replication sample (ADNI) are described in Table 1. The genetic information of 4,569 individuals (2,540 AD cases and 2,029 controls) was considered after passing QC thresholds based on MAF, HWE, MIND and GENO parameters calculated in PLINK. Additionally, we imputed a total of 1,231,704 and 1,245,964 QC-passing SNPs for the TGen1 and NIA-LOAD/NCRAD datasets, respectively. To account for bias still present after QC procedures, SNP association p-values were further assessed for genomic inflation, which is represented in Q-Q plots (Figure S1). All the datasets yielded an inflation factor (λ) between the acknowledged margins of 0.9 to 1.1, where the contribution of population structure to the genome-wide association is negligible [45]. Taking into account these considerations, we performed a meta-analysis using the inverse variance method, selecting p-values and ORs from the fixed effects model, assuming that these studies have been conducted under similar conditions and subjects [46]–[48]. The combined analysis showed a normal distribution of the p-values with an excess of significant signals seen only at the end of the curve, indicating likely true association events (λ = 1.05, Figure S1).

**Figure 2:**
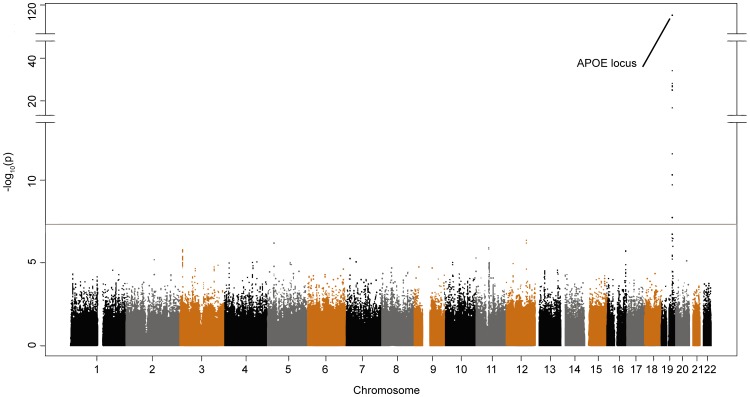
Genome wide meta-analysis results in AD. Manhattan plot showing the p-values obtained in the meta-analysis. The end and beginning of a chromosome is denoted by the change of color pattern of the SNPs (black, grey and brown dots). Genome-wide significance threshold is denoted by a red line (5.0×10^−8^). The Y-axis has been truncated to show all associated SNPs inside the APOE loci and to improve visualization of suggestive associations.

Whole-genome meta-analysis results are depicted as a Manhattan plot (Figure 2), with a significance threshold defined above log_10_ (5×10^−8^), which marks the beginning of genome-wide significant values [49]. In agreement with current reports, the strongest associations were located in a broad genomic region (>250,000 bp) in the vicinity of the APOE locus in chromosome 19 (Table 2). In particular, highly significant genome-wide associations signals were observed in the coding region of the translocase of the mitochondrial outer membrane gene (TOMM40: rs2075650, p = 8.54×10^−116^, OR = 4.48; rs157580, p = 9.6×10^−35^, OR = 0.51 and rs8106922, p = 1.17×10^−25^, OR = 0.57), upstream of the apolipoprotein C-I gene (APOC1: rs439401, p = 8.82×10^−29^, OR = 0.54), inside the poliovirus receptor related 2 isoform delta gene (PVRL2: rs6859, p = 7.87×10^−28^, OR = 1.7 and rs3852861 p = 5.32×10^−11^, OR = 0.64) and between TOMM40 and the APOE gene (rs405509, p = 2.29×10^−27^, OR = 0.57). In addition, in the same chromosomal region we observed genome-wide association, downstream of the basal cell adhesion molecule isoform 1 gene (BCAM: rs10402271, p = 1.98×10^−17^, OR = 1.46 and rs10405693, p = 2.83×10^−12^, OR = 1.49) and in the region of the B-cell CLL/lymphoma 3 gene (BCL3: rs8103315, p = 1.87×10^−8^, OR = 1.5). We note that the strength of the association signal for some SNPs is derived from the Pfizer and NIA-LOAD/NCRAD datasets only, since these were either not genotyped in the TGen1 sample or poorly imputed due to the intrinsic array density in that particular region of chromosome 19 in the Affimetrix GeneChip 500 K (See "N" column, Table 2). Interestingly, among the top 25 associations detected, novel genome-wide marginally significant signals outside chromosome 19 were observed: in chromosome 12 (intergenic: rs249153, p = 4.38×10^−07^, OR = 1.41; intergenic: rs249166 and rs249167, both with p = 6.91×10^−07^ and OR = 1.40) and in chromosome 5 (intergenic: rs13178362, p = 6.60×10^−07^, OR = 0.75), followed by trends inside the membrane-spanning 4-domains, subfamily A, member 3 gene in chromosome 11 (MS4A3: rs474951, p = 1.25×10^−6^, OR = 0.79 and rs528823, p = 1.55×10^−6^, OR = 0.79) and also within the Fanconi anemia group D2 gene in chromosome 3 (FANCD2: rs1552244, p = 1.63×10^−06^, OR = 0.76; rs9849434, p = 1.88×10^−06^, OR = 0.71).

**Table 2:** Meta-analysis 25 top hits.

Chr	BP	SNP	A1	A2	N	P	OR	Gene
19	50,087,459	rs2075650	G	A	3	8.54E-116	4.48	TOMM40, PVRL2
19	50,087,106	rs157580	G	A	2	9.60E-35	0.51	TOMM40, PVRL2
19	50,106,291	rs439401	T	C	2	8.82E-29	0.54	APOC1, APOE
19	50,073,874	rs6859	A	G	3	7.87E-28	1.70	PVRL2
19	50,100,676	rs405509	G	T	2	2.29E-27	0.57	TOMM40, APOE
19	50,093,506	rs8106922	G	A	2	1.17E-25	0.57	TOMM40
19	50,021,054	rs10402271	G	T	3	1.98E-17	1.46	BCAM
19	50,018,504	rs10405693	T	C	2	2.83E-12	1.49	BCAM
19	50,074,901	rs3852861	T	G	2	5.32E-11	0.64	PVRL2
19	49,857,752	rs714948	A	C	3	2.03E-10	1.60	PVR
19	49,946,008	rs8103315	A	C	2	1.87E-08	1.50	BCL3
19	50,054,507	rs377702	A	G	3	1.99E-07	1.29	PVRL2
19	49,933,947	rs2927438	A	G	2	3.32E-07	1.35	intergenic
19	50,421,115	rs346763	A	G	2	3.67E-07	1.80	EXOC3L2
12	93,848,520	rs249153	C	T	2	4.38E-07	1.41	intergenic
19	50,053,064	rs440277	A	G	2	4.79E-07	0.76	PVRL2
19	50,043,586	rs1871047	G	A	3	4.87E-07	0.79	PVRL2
5	30,214,770	rs13178362	C	T	3	6.60E-07	0.75	intergenic
12	93,852,604	rs249166	T	G	2	6.91E-07	1.40	intergenic
12	93,854,404	rs249167	A	T	2	6.91E-07	1.40	intergenic
19	50,417,946	rs10422797	C	T	2	1.02E-06	1.82	EXOC3L2
11	59,595,197	rs474951	G	T	3	1.25E-06	0.79	MS4A3
11	59,593,673	rs528823	T	C	3	1.55E-06	0.79	MS4A3
3	10,110,577	rs1552244	G	A	3	1.63E-06	0.76	FANCD2, FANCD2OS
3	10,108,710	rs9849434	A	G	2	1.88E-06	0.71	FANCD2, FANCD2OS

Chr: Chromosome; BP: Physical Position (Base Pair, NCBI 36.3/Hg18); A1: Allele 1 (Affected); A2: Allele 2 (Reference); N: Number of datasets with information; p-value: Fixed effect model p-value; OR: Odd Ratio.

### Pathway Analysis

To test the hypothesis that highly-connected sub-networks (SNs) enriched with minor associations might be significantly overrepresented in AD, we performed a gene-oriented pathway analysis by loading meta-analysis results into a high confidence functional protein association network (FPAN), gathered from the STRING database [32]. First, to avoid noise, the analysis was restricted to SNPs with p-value <0.05 (Table S1). Second, we calculated whole-gene association values using two alternative approaches: (i) the extraction of a gene-wise p-value, corresponding to the strongest association signal within a gene (Meta-GW) [17]; and (ii) the derivation of a more stringent gene-based p-value using the extended Simes test (Meta-GATES), which combines all association signals within a gene and controls for the bias that could be generated by gene-size or LD structure among markers [31]. Thus, we observed 66,204 SNP association p-values <0.05 tagging 7,527 genes. Of these, we loaded only 4,891 and 4,647 gene p-values (from GW and GATES procedures, respectively) into FPAN (Table S2), which was composed by 14,793 genes having 229,357 non-redundant high-confidence interactions (note that not all genes are informative in the FPAN). Third, we conducted the search for significant SNs in AD using the information from GW and GATES procedures in comparison with an expected background distribution among the FPAN created from 10,000 permutations (See Methods). Fourth, we repeated the search 10 times in order to explore whether the SNs structure (gene members), interactions and significance was consistent across iterations and not the result of the Monte Carlo procedure. As an example, we observed that the top SN1 had a 97.6% of concordance in gene structure and interactions (see also Table S3). Fifth, the above results were controlled by 2 further module searches, this time including permuted data or results without genome wide (WGW) significance (Figure 1). While in the former search, the p-values were permuted 10 times over the entire FPAN to determine if the SN structure and its score could be obtained by chance (Meta-GW-Permuted, Meta-GATES-Permuted); in the latter, we discarded the possibility that the SNs could be the result of bias due to the strong genome-wide significant p-values within the APOE locus.

**Figure 3:**
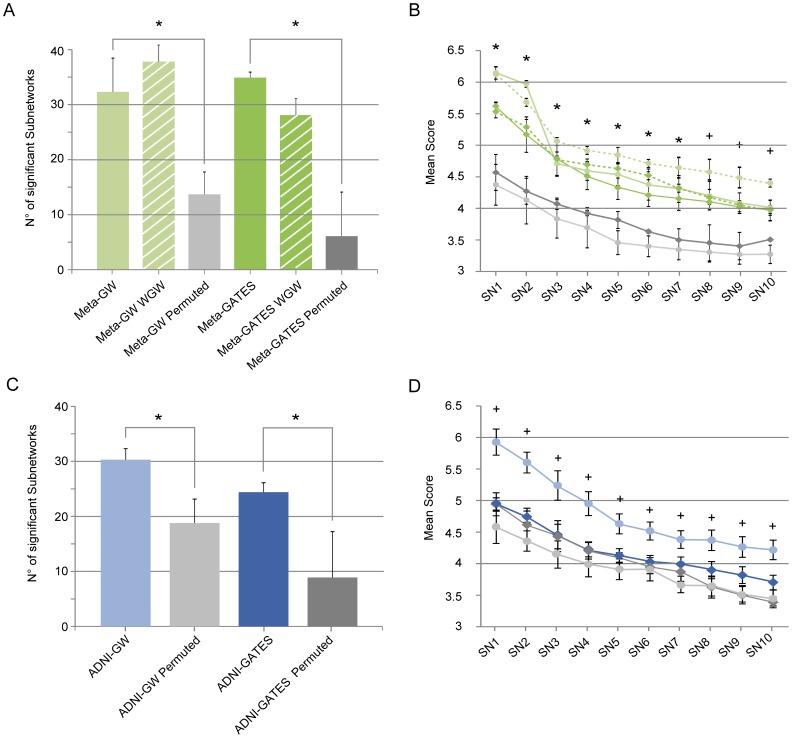
SN search results. (A) The number of significant SNs (size <50 and score>3) in Meta-GW (light green) and Meta-GATES (dark green) is shown compared with same values permuted across the FPAN: Meta-GW-Permuted (light grey) and Meta-GATES-Permuted (dark grey). (B) Score comparison of the top 10 SNs obtained in the corresponding module searches presented in (A). (C) The number of significant SNs in the replication step for ADNI-GW (light blue) and ADNI-GATES (dark blue) analysis, in comparison with their corresponding permuted controls: ADNI-GW-Permuted (light grey) and ADNI-GW-Permuted (dark grey). (D) Score comparison of the top 10 SNs obtained for each module searches presented in (C). Caped bar/points denote SD; Significant differences between real and permuted data observed in GW and GATES analysis are denoted by an asterisk and those between real and permuted data observed only in GW analysis are denoted by a plus sign (two-sided Student's t-test; p<0.01).

The result of the global search for overrepresented modules in AD is presented in Figure 3. First, the total number of significant SNs obtained in the Meta-GW analysis (average number  = 32.3, SD = 6.14) was significantly higher (p = 2.51×10^−7^), than those arising from chance (Figure 3A; Meta-GW Permuted; average = 13.7, SD = 4.05). Likewise, the total number of significant SNs in Meta-GATES was similar to Meta-GW (average number  = 34.9, SD = 0.99) and significantly higher (p = 1.35×10^−9^) than those obtained by chance (Figure 3A; Meta-GATES Permuted; average  = 6.1, SD = 7.99). Second, the SN scores in each procedure were always significantly higher among real vs. permuted data (Figure 3B). Third, the number and scores of the modules obtained in the WGW control was similar to the ones obtained with the whole set of associations, indicating that the strongest associations of the meta-analysis did not influence the present observations (Figure 3A and B). Fourth, the modules obtained in the Meta-GW and Meta-GATES searches, remained consistent in significance and structure across iterations, changing only in their respective rank/score. Remarkably, the most significant SN detected in each approach (Meta-GW SN1: S = 6.14, p-value  = 8.16×10^−8^; and Meta-GATES SN1: S  = 5.62, p-value  = 1.77×10^−5^; Figure 3B and Table 3) was identical in structure differing only in the presence of the guanine nucleotide binding protein (G protein) alpha z polypeptide gene (GNAZ), which was absent in Meta-GATES SN1 (Table S4). Finally, we note that the list of genes contained in each SN was not replicated in the permutation analyses and that only 2 out of 688 permuted genes were also seen either in Meta-GW SN1 or Meta-GATES SN1 (Figure S2). Altogether, these results indicate that the quantity, significance and structure of the modules identified could not be reached by chance, strongly suggesting that these sub-networks could be biologically meaningful in the etiology of AD.

**Table 3:** GO terms enriched in Meta-GW and Meta-GATES top 3 Sub-Networks.

SN		GO ID	GO Name	GISN	GIP	TG	p-value
**Meta-GW**							
SN1	BP	GO:0007215	glutamate receptor signaling pathway	11	46	49	2.67E-11
		GO:0007610	behavior	17	497	49	1.02E-08
		GO:0003001	generation of a signal involved in cell-cell signaling	12	332	49	1.03E-08
	CC	GO:0045202	synapse	21	461	49	2.87E-18
		GO:0044456	synapse part	19	347	49	5.90E-18
		GO:0031234	extrinsic to internal side of plasma membrane	9	50	49	2.13E-13
	MF	GO:0008066	glutamate receptor activity	13	24	49	1.21E-18
		GO:0035254	glutamate receptor binding	8	22	49	2.03E-10
		GO:0031683	G-protein beta/gamma-subunit complex binding	8	22	49	4.63E-10
SN2	BP	GO:0007411	axon guidance	33	341	46	1.20E-09
		GO:0040012	regulation of locomotion	14	435	46	1.16E-07
		GO:0035385	Roundabout signaling pathway	3	3	46	3.81E-07
	CC	GO:0031252	cell leading edge	12	245	46	9.35E-11
		GO:0005955	calcineurin complex	3	4	46	1.69E-07
		GO:0031256	leading edge membrane	8	87	46	4.67E-07
	MF	GO:0005042	netrin receptor activity	4	4	46	6.30E-10
		GO:0048495	Roundabout binding	3	4	46	6.77E-08
SN3	BP	GO:0035385	Roundabout signaling pathway	3	3	45	1.60E-07
		GO:0061364	apoptotic process involved in luteolysis	3	3	45	1.74E-07
		GO:0021889	olfactory bulb interneuron differentiation	4	11	45	3.15E-06
	MF	GO:0048495	Roundabout binding	3	4	45	9.46E-07
**Meta-GATES**							
SN1	BP	GO:0007215	glutamate receptor signaling pathway	11	46	48	1.86E-11
		GO:0007610	behavior	17	497	48	6.58E-09
		GO:0003001	generation of a signal involved in cell-cell signaling	12	332	48	7.92E-09
	CC	GO:0045202	synapse	21	461	48	1.69E-18
		GO:0044456	synapse part	19	347	48	3.70E-18
		GO:0097060	synaptic membrane	16	202	48	2.55E-13
	MF	GO:0008066	glutamate receptor activity	13	24	48	1.21E-18
		GO:0035254	glutamate receptor binding	8	22	48	1.14E-10
		GO:0031683	G-protein beta/gamma-subunit complex binding	7	22	48	8.22E-09
SN2	BP	GO:0008202	steroid metabolic process	11	269	50	2.13E-09
		GO:0010876	lipid localization	13	221	50	3.81E-09
		GO:0006869	lipid transport	12	195	50	8.12E-09
	CC	GO:0032994	protein-lipid complex	5	36	50	2.51E-07
	MF	GO:0048495	Roundabout binding	3	4	50	2.02E-06
		GO:0071814	protein-lipid complex binding	4	23	50	3.22E-06
		GO:1901681	sulfur compound binding	7	163	50	8.26E-06
SN3	BP	GO:0046777	protein autophosphorylation	16	174	21	2.30E-12
		GO:0040012	regulation of locomotion	10	435	21	6.55E-08
		GO:0051270	regulation of cellular component movement	10	443	21	1.14E-07
	CC	GO:0045202	synapse	7	461	21	3.28E-06
	MF	GO:0019199	transmembrane receptor protein kinase activity	12	82	21	1.18E-12
		GO:0019838	growth factor binding	9	99	21	3.42E-12
		GO:0004713	protein tyrosine kinase activity	16	136	21	1.09E-10

SN: Sub-network; GO ID:Gene ontology term ID; GIP: Genes in population; GISN: Genes in sub-network; TG: Total genes in SN; BP: Biological process; CC:Cellular component; MF: Molecular function.

### Glutamate signaling is overrepresented in AD

To examine the above-mentioned hypothesis, gene ontology (GO) term enrichment was assessed in the top 10 SNs identified using the package Ontologizer (see Methods). Table 3 presents the top 3 Meta-GW and Meta-GATES SNs as a function of biological process (BP), cellular component (CC) and molecular function (MF) categories. Meta-GW results indicated that: SN1 was heavily composed by genes acting at the synapse (21/461, p = 2.87×10^−18^), participating in the glutamate receptor signaling pathway (11/46, 2.67×10^−11^) and specifically related to glutamate receptor activity (13/24, p = 1.21×10^−18^); SN2 was mostly over-represented by genes belonging to the axon guidance biological process (33/341, p = 1.20×10^−9^), located mostly at the cell leading edge (12/245, p = 9.35×10^−11^); and SN3 was over-represented by the roundabout signaling pathway (3/3, p = 1.6×10^−7^). On the other hand, the results with the alternative and more stringent Meta-GATES procedure showed that SN1 had identical ontological enrichment patterns as observed for Meta-GW SN1, being glutamate receptor activity the most significant category (13/24, 1.69×10^−18^). Interestingly, Meta-GATES SN2 contained several genes involved in lipid metabolism including categories such as steroid metabolic process and lipid localization (11/269, p = 2.13×10^−9^ and 13/221, p = 3.81×10^−9^, respectively). Finally, Meta-GATES SN3 was composed of genes participating in transmembrane receptor protein kinase activity (12/82, p = 1.18×10^−12^), growth factor binding (9/99, p = 3.42×10^−12^), protein autophosphorylation (16/174, 2.30×10^−12^) and located mainly at the synapse (7/461, 3.28×10^−6^). Specific SNs features and components are described in Table S4. The complete set of ontologies overrepresented in the first top 10 SNs (SN1-SN10) is provided in Table S5.

### Replication of glutamate signaling in the ADNI dataset

Considering that glutamate signaling pathway components were consistently present in significant SNs enriched with minor associations to AD, both in the Meta-GW and Meta-GATES analyses, and since both procedures were originated from a single set of SNP associations, we next interrogated the ADNI dataset under the same pipeline (Figure 1), as an attempt to replicate the results in an independent sample of AD individuals. This additional dataset was composed of 693 subjects of which 499 were cases and 194 were controls (Table 1). Genetic association values were calculated replicating the quantitative trait locus (QTL) method reported in the original study [26], which is based on the composite memory score, a measure of the level of memory impairment, reported for each patient (see Methods). Although, the ADNI case cohort includes subjects with mild cognitive impairment (MCI), the phenotype is considered a transitional state with significant risk of progression to clinically diagnostic AD [50], which validates their inclusion. After the corresponding QC procedures, the ADNI dataset showed no significant genomic inflation (λ = 1.02, Figure S1). According to was described in the original publication, our results indicate that QTL testing yielded 25,785 SNP associations (p-value <0.05), tagging 4,915 genes (Table S6), did not reach genome wide significant levels. Marginal associations were observed within the dual specificity phosphatase 23 (DUSP23) gene in chromosome 1 (rs1129923, p = 1.07×10^−6^), the 3'-phosphoadenosine 5'-phosphosulfate synthase 1 (PAPSS1) gene (rs9569, p = 6.84×10^−6^) and in the phosphatidylinositol-4-phosphate 3-kinase, catalytic subunit type 2 gamma (PIK3C2G) gene (rs10841025, p = 9.01×10^−6^), as well as association signals in intergenic regions on chromosome 17 and 3 (rs9890008, p = 4.25×10^−6^ and rs4857008, p = 5.88×10^−6^, respectively).

We introduced 3,244 and 3,113 p-values, ADNI-GW and ADNI-GATES respectively (Table S2), into the same FPAN used for the Meta-analysis and module search was carried out with their respective null datasets (ADNI-GW-Permuted, ADNI-GATES-Permuted), since in the absence of genome wide significant results, the WGW control was not necessary. In general agreement with the meta-analysis data, global results indicated that the number of significant modules obtained in either ADNI-GW (average number  = 30.3, SD = 2.00) or ADNI-GATES (average number  = 24.4, SD = 1.7), was significantly higher (p = 7.01×10^−3^ and p = 1.80×10^−5^, respectively), than those obtained by chance (Permuted; Figure 3C). Interestingly, when comparing the scores of the first 10 SNs only the ones belonging to the ADNI-GW analysis remained significantly above their respective permuted ones (Figure 3D) and thus were considered for further analysis (Table S4).

GO term enrichment in ADNI indicated that genes belonging to categories such as voltage-gated calcium channel complex and ion channel complex were significantly overrepresented in AD (p = 1.24×10^−8^ and p = 9.24×10^−7^, respectively; see also Table S4 and Table S5 for complete ontological results). Moreover, we replicated multiple modules enriched with glutamate signaling genes (Table 4), including modules SN3 (S = 5.23, p = 5.09×10^−8^), SN4 (S = 4.94, p = 7.14×10^−8^) and SN7 (S = 4.38, p-value  = 3.40×10^−8^). Individual sub-network structure is presented in Figure S3.

**Table 4:** Glutamate positive sub-networks in ADNI-GW analysis.

SN	Type	GO ID	GO Name	GISN	GIP	TG	p-value
SN3	BP	GO:0035637	multicellular organismal signaling	19	483	48	5.87E-12
		GO:0019226	transmission of nerve impulse	19	467	48	6.22E-10
		GO:0007215	glutamate receptor signaling pathway	7	44	48	5.09E-08
	CC	GO:0044447	axoneme part	6	20	48	9.54E-11
		GO:0044463	cell projection part	12	235	48	1.12E-10
		GO:0008328	ionotropic glutamate receptor complex	6	21	48	3.24E-08
	MF	GO:0008066	glutamate receptor activity	5	19	48	1.78E-06
SN4	BP	GO:0031280	Neg. regulation of cyclase activity	4	19	22	2.59E-06
		GO:0051350	Neg. regulation of lyase activity	4	20	22	3.17E-06
		GO:0030809	Neg. regulation of nucleotide biosynthetic process	4	23	22	2.38E-06
	MF	GO:0008066	glutamate receptor activity	6	19	22	7.14E-08
SN7	BP	GO:0007215	glutamate receptor signaling pathway	7	44	40	5.09E-08
	CC	GO:0008328	ionotropic glutamate receptor complex	4	21	40	2.09E-05
	MF	GO:0008066	glutamate receptor activity	5	19	40	3.40E-08

SN: Sub-network; GO ID: Gene ontology term ID; GIP: Genes in population; GISN: Genes in sub-network; TG: Total genes in SN; BP: Biological process; CC: Cellular component; MF: Molecular function.

### KEGG pathway enrichment

**Figure 4:**
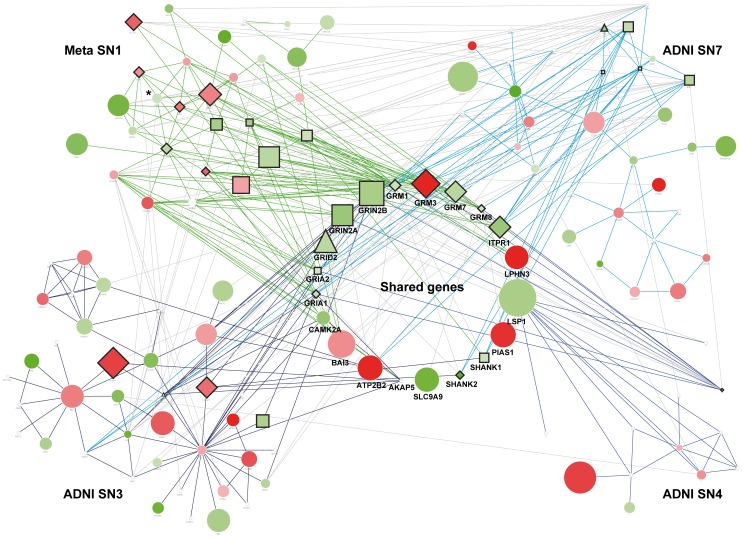
Structure and relationships between glutamate signaling SNs overrepresented in AD. SN gene composition (nodes) and interactions (edges) are shown for: Meta-SN1 in the upper left corner with green edges (which includes GW and GATES modules, GNAZ* gene only present in GW); ADNI-GW SN3 in the bottom left corner with dark blue edges; ADNI-GW SN4 in the bottom right corner with blue edges and ADNI-GW SN7 in the upper right corner with light blue edges. Genes shared by at least 2 SNs are located at the center in bold font and cross interactions between genes inside each module are denoted by light grey edges. Node color represents the OR behavior in a gradient from green to red values (i.e. green: OR<1; red OR>1; white: OR = 1), denoting protection and risk, respectively. Similarly, node size is proportional to the –log10 p-value obtained from the meta-analysis (if absent, node size is the minimum). Triangle shaped nodes marks genes belonging to the glutamate signaling pathway GO term (GO:0007215); Diamond shaped nodes denotes genes belonging to KEGG Glutamatergic synapse pathway (hsa04724); Squares Square shaped nodes denotes genes belonging to both gene ontology term GO:0007215 and KEGG hsa04724 pathway.

**Figure 5:**
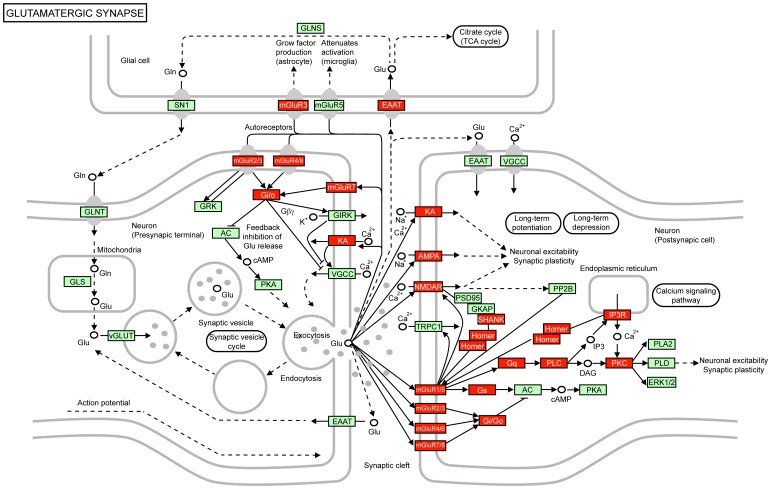
Overrepresented glutamate signaling components in the functional synapse. The original version of the hsa04724 KEGG pathway (Glutamatergic synapse) is shown. Black arrows denote direct molecular interaction or relation between gene products (green squares) or other types of molecules (unfilled circles), while black arrows with dashed lines denote an indirect effect between the each node. The relationship with other KEGG pathways is shown with the presence of white round rectangles. Gene symbols in components belonging to Meta-GW and META-GATES SN1 are denoted in red.

KEGG provided information regarding 280 pathways involving 6,733 genes. Although in comparison with the GO database the amount of information provided by KEGG is substantially reduced, the fact that each annotation is manually curated makes any association much more reliable [51]. Notably, throughout this analysis we detected that glutamate signaling was again the main overrepresented biological process in both Meta-GW SN1 (p = 1.10×10^−28^) and Meta-GATES SN1 (p = 5.94×10^−29^) sub-networks (Table S7). Glutamatergic synapse, as a KEGG pathway category, was also significantly associated in ADNI sub-networks SN3, SN4 and SN7 (p = 1.20×10^−06^, p = 1.32×10^−06^ and p = 8.18×10^−10^, respectively; Table S7). Finally, logical and structural relationships of all sub-networks enriched with glutamate signaling genes from the meta-analysis and the ADNI-replication dataset allowed us to define a list of genes of interest, which were shared at least by 3 SNs (Figure 4). The list was composed by 20 signaling components, including membrane-anchored ionotropic (GRIN2A, GRIN2B, GRID2, GRIA1 and GRIA2) and metabotropic glutamate receptors (GRM1, GRM3, GRM7 and GRM8), intracellular downstream effectors CAMK2A and AKAP5, as well as scaffold proteins SHANK1 and SHANK2, which are required for proper formation and function of neuronal synapses [52]. The functional relationship of these signaling components in the context of a glutamatergic synapse is shown in Figure 5.

### Expression of glutamate-signaling genes in the human brain

**Figure 6:**
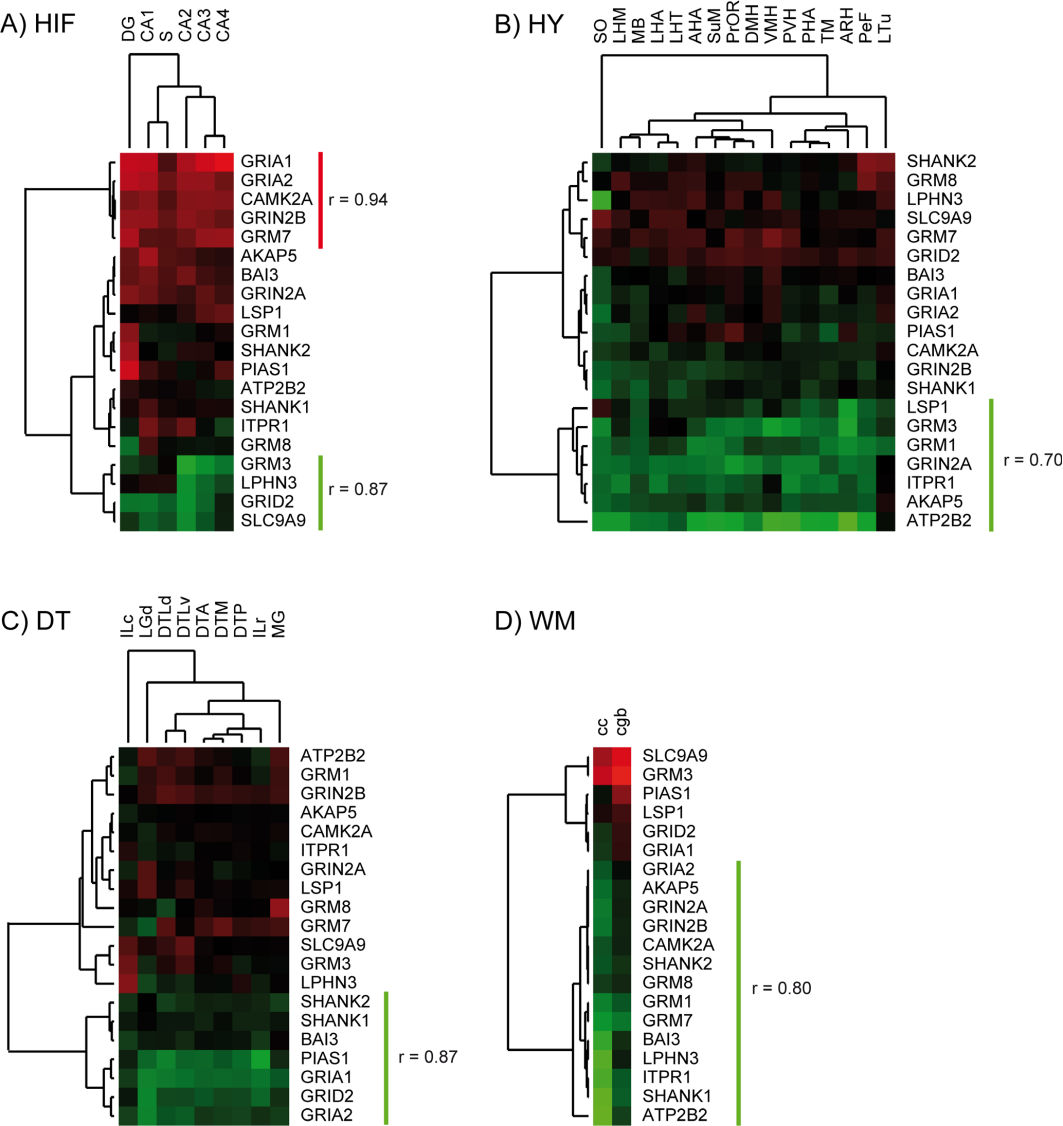
Gene expression analysis of glutamate signaling components in selected human brain regions. Heatmap and dendrogram of normalized expression levels of the 20 genes of interest displaying significant clustering in: (A) hippocampal formation (HIF); (B) hypothalamus (HY); (C) Dorsal Thalamus (DT); and (D) white matter (WM). Heatmaps were generated using normalized Z score gene-wise expression values, which were averaged from 6 brain donor individuals (ids. H0351.2001, H0351.2002, H0351.1009, H0351.1012, H0351.1015 and H0351.1016). Bright red and green color indicates high (Z>2) and low expression (Z<2). Highly correlated gene clusters (Euclidean distance correlation coefficient r>0.7) are denoted by colored lines in the dendrograms: green clusters, indicates low expression patterns; red clusters show high levels of expression of correlated genes. Gene expression patterns in the corresponding substructures are shown for HIF: Dentate Gyrus (DG); Cornu Ammonis 1 (CA1); Cornu Ammonis 2 (CA2); Cornu Ammonis 3 (CA3); Cornu Ammonis 4 (CA4) and Subiculum (S). For HY: Anterior Hypothalamic Area (AHA); Lateral hypothalamic Area (LHA); Paraventricular Nucleus of the Hypothalamus (PVH); Supraoptic Nucleus (SO); Lateral Hypothalamic Area, Mammillary Region (LHM); Mammillary Body (MB); Posterior Hypothalamic Area (PHA); Supramammillary Nucleus (SuM); Tuberomammillary Nucleus (TM); Preoptic Region (PrOR); Arcuate Nucleus of the Hypothalamus (ARH); Dorsomedial Hypothalamic Nucleus (DMH); Lateral Hypothalamic Area, Tuberal Region (LHT); Lateral Tuberal Nucleus (LTu); Perifornical Nucleus (PeF); Ventromedial Hypothalamic Nucleus (VMH). For DT: Anterior Group of Nuclei (DTA); Caudal Group of intralaminar Nuclei (ILc); Dorsal Lateral Geneiculate Nucleus (LGd); Lateral Group of Nuclei, Dorsal Division (DTLd); Lateral Group of Nuclei, Ventral Division (DTLv); Medial Geniculate Complex (MG); Medial Group of Nuclei (DTM); Posterior Group of Nuclei (DTP); Rostral Group of Intralaminar Nuclei (ILr). For WM: Cc: Corpus callosum; Cgb: Cingulum bundle.

At the physiological level, to explore if there was a transcriptional relationship among the glutamate signaling genes previously identified, we examined their expression profiles in 27 normal human brain regions, using the information from the Allen Brain Atlas [42], as a reference. Clearly, the expression pattern of these components clustered in brain regions tightly related to AD pathology, such as the hippocampal formation, hypothalamus and white matter [53]–[55] (Figure 6). While it is known that glutamate signaling is active in these brain domains [56]–[58], it was interesting to find out clusters with high- and low-expression levels. For instance, GRIA1, GRIA2, CAMK2A, GRIN2B and GRM7 were found among highly expressed gene clusters in the hippocampal formation (r = 0.94), particularly in the CA2–CA3 and CA4 region (Figure 6A), while GRM3, LPHN3, GRID2 and SLC9A9 were grouped in a low-expression cluster (r = 0.87). Low-expression clusters were also observed in the hypothalamus (LSP1, GRM3, GRM1, GRIN2A, ITPR1, AKAP5 and ATP2B2; Figure 6B), the dorsal thalamus (SHANK2, SHANK1, BAI3, PIAS1, GRIA1, GRID2 and GRIA2; Figure 6C) and also distinguished in the white matter (GRIA2, AKAP5, GRIN2A, GRIN2B, CAMK2A, SHANK2, GRM8, GRM1, GRM7, BAI3, LPHN3, ITPR1, SHANK1 and ATP2B2; Figure 6D). Interestingly, there was an inverse relationship in the expression pattern of a subset of these genes, since components highly expressed in the hippocampus were found in low-expression clusters in the white matter, and vice versa (i.e. GRIA2, CAMK2 and GRIN2B vs. GRM3 and SLC9A9). Altogether these results indicate that glutamate signaling components are differentially expressed in restricted brain domains for proper neuronal or glial functional activity (see also Figure 5).

## Discussion

In agreement with previous GWAS in AD [8], [13]–[16], [19] our meta-analysis detected strong genome-wide association signals in a 250 kb window of chromosome 19, centered in the coding/regulatory region of the TOMM40 gene, in close proximity to the APOE locus, and that also included significant signals in the PVRL2, APOC1, BCAM and BCL3 genes. While it has been suggested that the association of such extended region may reflect that other variants in LD with APOE may be of pathogenic importance, particularly a poly-T track in the TOMM40 gene [59], [60], recent studies have shown that APOE alleles account for essentially all the inherited risk of AD associated in this region [61]. Besides the signal in chromosome 19, we detected marginal associations of 2 novel SNPs in the MS4A3 gene, located in a wide LD region containing a cluster of SNPs in the MS4A6A/MS4A4E loci in the long arm of chromosome 11 (i.e. rs610932, rs670139, rs1562990, rs4938933 and rs983392), which reached genome-wide significant levels by other recent studies [16], [62], [63].

Assuming that the APOE locus is the major genetic hallmark associated with the disease and that it does not explain the entire susceptibility of AD [1], [7]–[9], we conducted a network-based pathway analysis with our meta-analysis results to explore the biology behind variants with minor effect size. Initially, to integrate the whole genetic contribution from the meta-analysis we used a gene-wise p-value (GW, single min p-value method) that has been widely applied in detecting novel associations using GWAS data [17], [64]. Although this approach has a certain bias for pathways enriched with larger genes and does not consider intergenic associations or LD structure, we note that the random permutation of p-values yielded a distribution of results expected by chance from where the actual data could be compared. Likewise, with the search for significant sub-networks with real and permuted data, and additionally with the WGW control, we believe that the actual contribution of the aforementioned problems to the final result is strongly surpassed by the combination of true minor effect size variants. Still, we considered appropriated the introduction of the GATES procedure that is specifically designed to directly address the gene size and LD structure issues and thus we ended up with a more stringent gene-oriented p-value. Notably, through this approach we replicated essentially the same SN (i.e. GW and GATES SN1), which was populated by genes related to glutamate signaling, differing only in the absence of GNAZ gene whose only association in the meta-analysis (Table S1; rs4820537, p = 0.02096) was found not informative in the GATES procedure. Glutamate signaling was further replicated in the ADNI dataset and this time it reached significant association levels in three SNs (ADNI-GW SN3, SN4 and SN7).

From a biological point of view, the relationship of these genetic observations with current knowledge about AD is straightforward. Glutamate signaling has been reported to regulate multiple biological processes, including fast excitatory synaptic transmission, neuronal growth and differentiation, synaptic plasticity, learning and memory [65], [66]. Degenerating neurons and synapses in AD brains are usually located within regions that project to or from areas displaying high densities of Aβ plaques and tangles [67] and in this regard, glutamatergic neurons located in the hippocampus, as well as in other areas of the brain, are severely affected by these neurotoxic insults [65], [67]. Likewise, it has been established that there is a relationship between glutamate receptor signaling and soluble Aβ oligomers in the hippocampus, affecting their expression and recycling, which leads to long term depression, synaptic loss and ultimately to cognitive deficit [68], [69]. Moreover, sustained activation of glutamate receptors at the synapse rise Ca^2+^ influxes and second messenger levels activating neuronal nitric oxide synthase (NO), increasing reactive nitrogen and oxygen species, thus contributing to neuronal damage independently of the presence of Aβ oligomers [70]. Alternatively, and from a genomic perspective, here we provide strong evidence that common genetic variants within a complete set of genes acting as ionotropic/metabotropic glutamate receptors and its downstream effectors are associated in a network context with AD. Accordingly, it has been recently reported that pathways related to neurotransmitter receptor-mediated calcium signaling and long-term potentiation are similarly associated with mild cognitive impairment and AD [26]. In addition we have found that the expression pattern of these glutamate signaling genes cluster in specific brain regions, which are affected during the development of the disease, such as the hippocampal formation and the hypothalamus. Therefore, it will be interesting to learn if the genes identified through our network-based pathway approach are spatially and coordinately modulated at the transcriptional or post-transcriptional level as a result of various trophic or toxic stimuli. Finally, our data extends the notion that the remaining genetic risk for complex traits, such as AD, is likely explained by the accumulation of functional genetic variants inside an entire pathway, rather than by punctual independent mutations.

## Supporting Information

Figure S1 **Quantile-Quantile (Q-Q) plots for GWAS datasets and combined meta-analysis.** Comparison of the association results for each SNP (black dots) with those expected by chance (red line) in TGen1 (A), NIA-LOAD/NCRAD (B), Pfizer (C) the final meta-analysis (D) and in the ADNI replication dataset. In each dataset, the genomic inflation factor (λ) is shown. Values of λ between 0.9 and 1.1 are considered unbiased by the population structure.
(TIF)

Figure S2 **Gene structure comparison between modules detected with real and permuted data.** Gene coincidences between Meta-GW SN1 (49 genes, light grey circle) and Meta-GATES SN1 (48 genes, dark grey circle) are shown in a Venn diagram and compared with the total number of genes in the first 10 modules of each permuted analysis: Meta-GW SN1 to SN10 (397 genes, light grey circle) and Meta-GATES SN1 to SN10 (354 genes, dark grey circle).
(TIF)

Figure S3 **Glutamate signaling SNs overrepresented in AD.** Meta-GW SN1 in conjunction with Meta-GATES SN1, and ADNI-GW SN3, ADNI-GW SN4, ADNI-GW SN7 sub-networks are shown in A through D, respectively. Nodes represent genes and edges their corresponding interactions extracted from FPAN based upon the information in the STRING database. Network legend is provided at the bottom panel: the node color represents the OR behavior in a gradient from green to red values (i.e. green: OR<1; red OR>1; white: OR = 1), denoting protection and risk, respectively. Similarly, node size and edge thickness are proportional to the -log10 p-value obtained in the meta-analysis (if absent, node size is the minimum) and the combined score of interaction. Asterisk in GNAZ gene is a reminder that this gene is only present in Meta-GW SN1.
(TIF)

Table S1 **Meta-analysis associations (p-value <0,05) with additional annotations.**
(XLSX)

Table S2 **Gene-wise and GATES p-values introduced to FPAN in the Meta and ADNI analyses.**
(XLSX)

Table S3 **Gene structure concordance for the main sub-network (SN1) across module search iterations.**
(XLSX)

Table S4 **Top 10 Sub-networks main features and components.**
(XLSX)

Table S5 **Gene Ontologies terms overrepresented in Meta and ADNI Top 10 Sub-networks.**
(XLSX)

Table S6 **ADNI Associations with additional annotations.**
(XLSX)

Table S7 **KEGG pathways overrepresented in Meta and ADNI Top 10 Sub-networks.**
(XLSX)

Checklist S1 **PRISMA Checklist.**
(PDF)
