# Supplementary figures and images for: Overrepresentation of Glutamate Signaling in Alzheimer's Disease: Network-Based Pathway Enrichment Using Meta-Analysis of Genome-Wide Association Studies
Source: PLoS One. 2014 Apr 22;9(4):e95413. doi: 10.1371/journal.pone.0095413 (PMC3995778; doi:10.1371/journal.pone.0095413)

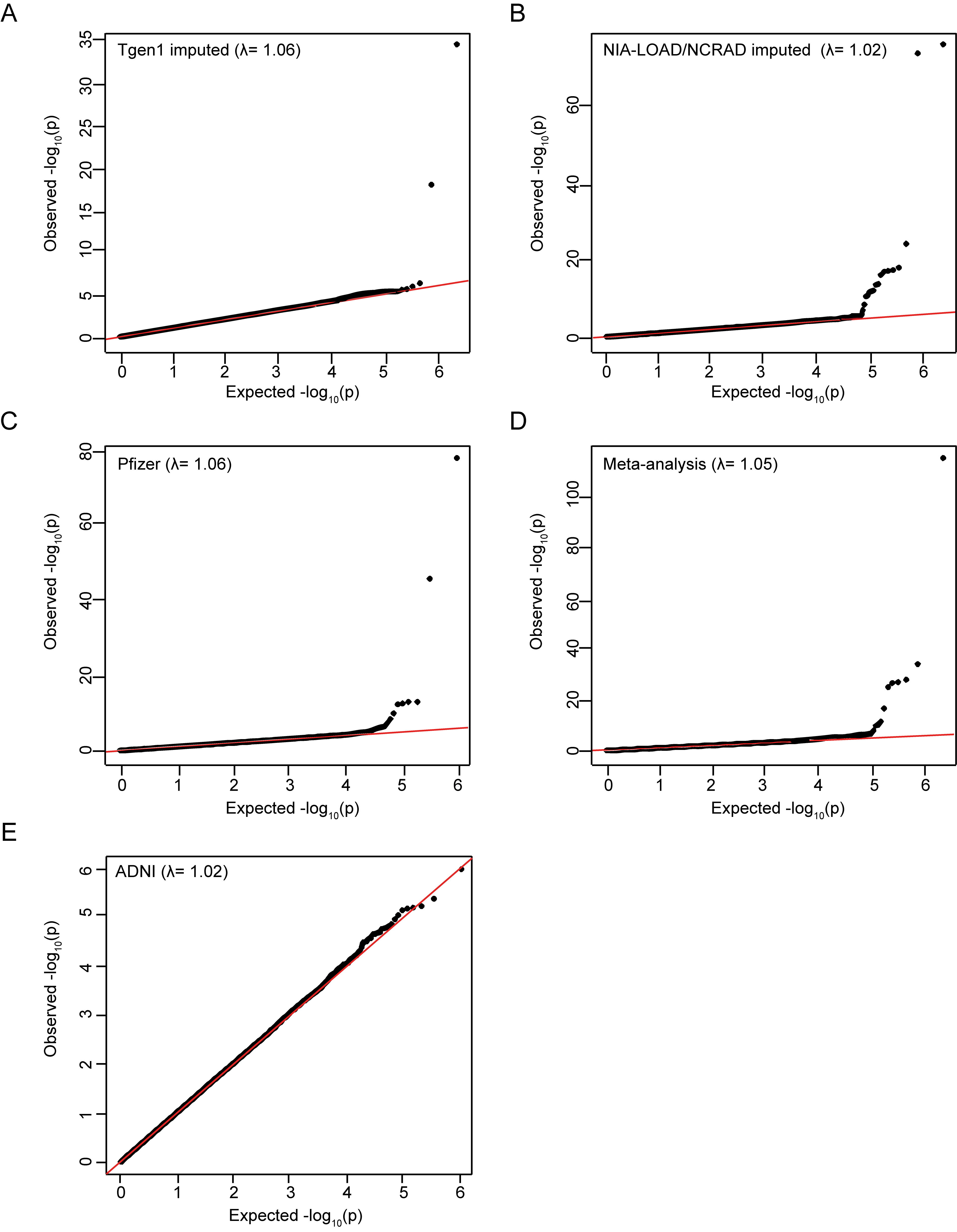

Supplement: Figure S1 — Quantile-Quantile (Q-Q) plots for GWAS datasets and combined meta-analysis. Comparison of the association results for each SNP (black dots) with those expected by chance (red line) in TGen1 (A), NIA-LOAD/NCRAD (B), Pfizer (C) the final meta-analysis (D) and in the ADNI replication dataset. In each dataset, the genomic inflation factor (λ) is shown. Values of λ between 0.9 and 1.1 are considered unbiased by the population structure. (TIF) [file pone.0095413.s001.tif]

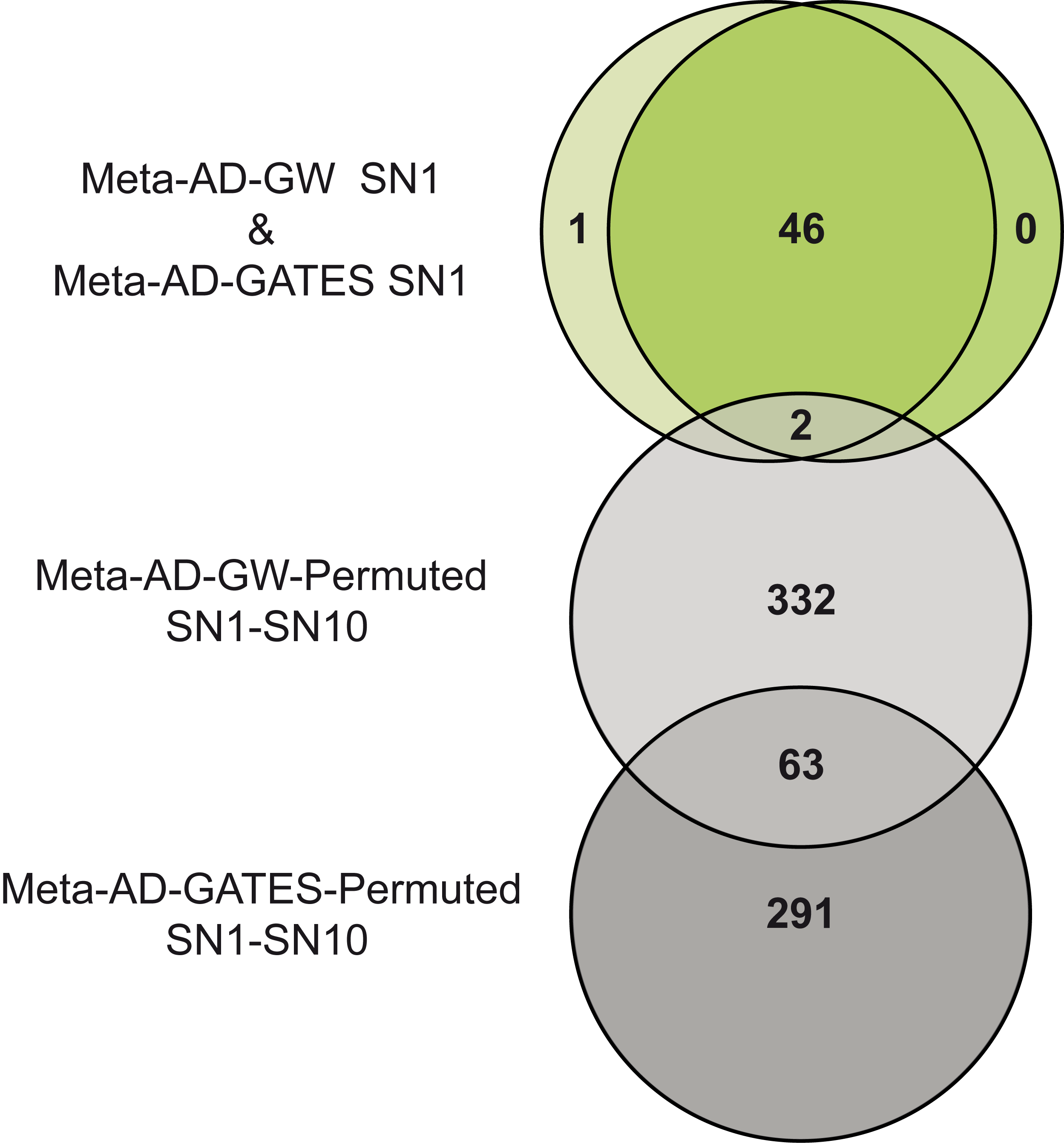

Supplement: Figure S2 — Gene structure comparison between modules detected with real and permuted data. Gene coincidences between Meta-GW SN1 (49 genes, light grey circle) and Meta-GATES SN1 (48 genes, dark grey circle) are shown in a Venn diagram and compared with the total number of genes in the first 10 modules of each permuted analysis: Meta-GW SN1 to SN10 (397 genes, light grey circle) and Meta-GATES SN1 to SN10 (354 genes, dark grey circle). (TIF) [file pone.0095413.s002.tif]

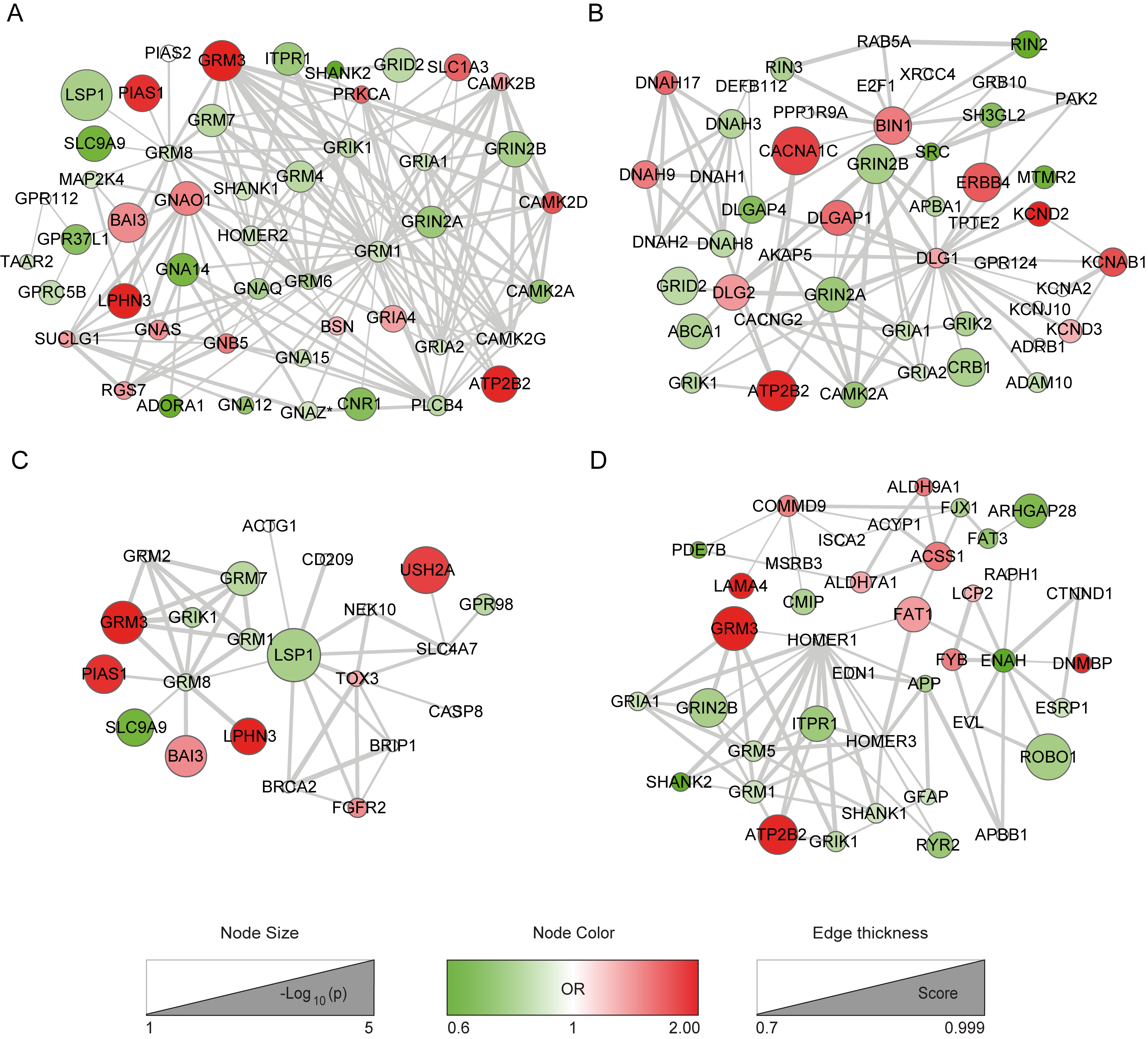

Supplement: Figure S3 — Glutamate signaling SNs overrepresented in AD. Meta-GW SN1 in conjunction with Meta-GATES SN1, and ADNI-GW SN3, ADNI-GW SN4, ADNI-GW SN7 sub-networks are shown in A through D, respectively. Nodes represent genes and edges their corresponding interactions extracted from FPAN based upon the information in the STRING database. Network legend is provided at the bottom panel: the node color represents the OR behavior in a gradient from green to red values (i.e. green: OR<1; red OR>1; white: OR = 1), denoting protection and risk, respectively. Similarly, node size and edge thickness are proportional to the -log10 p-value obtained in the meta-analysis (if absent, node size is the minimum) and the combined score of interaction. Asterisk in GNAZ gene is a reminder that this gene is only present in Meta-GW SN1. (TIF) [file pone.0095413.s003.tif]
